# Aqua­(4-cyano­pyridine-κ*N* ^4^)(5,10,15,20-tetra­phenyl­porphyrinato-κ^4^ *N*)magnesium

**DOI:** 10.1107/S1600536812049434

**Published:** 2012-12-08

**Authors:** Khaireddine Ezzayani, Mohamed Salah Belkhiria, Shabir Najmudin, Cecilia Bonifácio, Habib Nasri

**Affiliations:** a Laboratoire de Physico-chimie des Matériaux, Université de Monastir, Faculté des Sciences de Monastir, Avenue de l’Environnement, 5019 Monastir, Tunisia; b Faculdade de Medicina, Veterinària, Universidade Tecnica de Lisboa, Avenida da Universidade Tecnica, 1300-477 Lisboa, Portugal; c REQUIMTE/CQFB Departamento de Quimica, Faculdade de Ciencias e Tecnologia, Universidade Nova de Lisboa, 2829-516 Caparica, Portugal

## Abstract

In the title complex, [Mg(C_44_H_28_N_4_)(C_6_H_4_N_2_)(H_2_O)], the Mg^2+^ cation is octa­hedrally coordinated and lies on an inversion center with the axially located 4-cyano­pyridine and aqua ligands exhibiting 50% substitutional disorder. The cyano-bound 4-cyano­pyridine mol­ecule also is disordered across the inversion centre. The four N atoms of the pyrrole rings of the dianionic 5,10,15,20-tetra­phenyl­porphyrin ligand occupy the equatorial sites of the octa­hedron [Mg—N = 2.0552 (10) and 2.0678 (11) Å] and the axial Mg—(N,O) bond length is 2.3798 (12) Å. The crystal packing is stabilized by weak inter­molecular C—H⋯π inter­actions.

## Related literature

For general background to magnesium porphyrin species and their applications, see: Ghosh *et al.* (2010 ▶). For the synthesis of the [Mg(TPP)(H_2_O)] (TPP is tetraphenylporphyrin) complex, see: Timkovich & Tulinsky (1969 ▶). For related structures, see: Choon *et al.* (1986 ▶); Imaz *et al.* (2005 ▶); Hibbs *et al.* (2003 ▶); Etkin *et al.* (1998 ▶); Yang *et al.* (2008 ▶). For a description of the Cambridge Structural Database, see: Allen (2002 ▶).
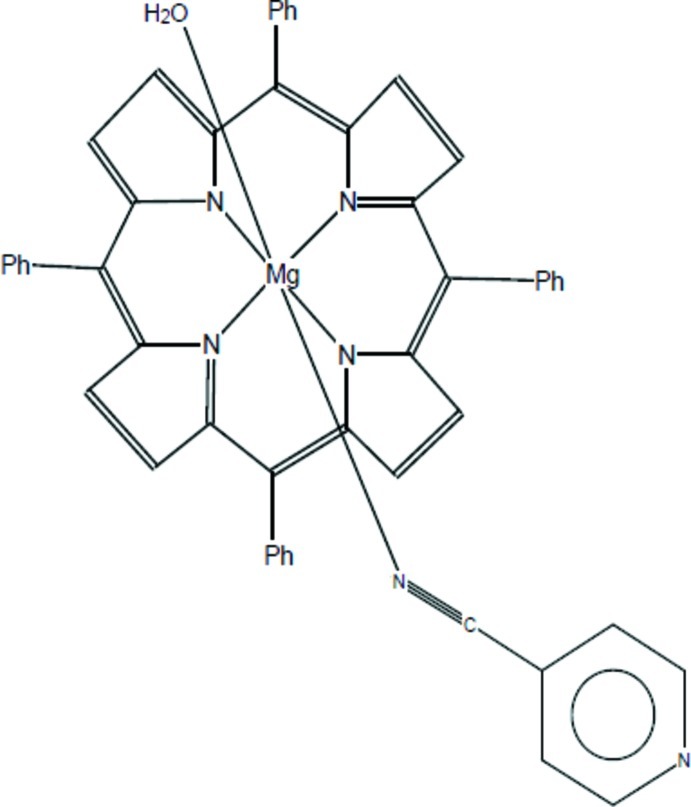


## Experimental

### 

#### Crystal data

[Mg(C_44_H_28_N_4_)(C_6_H_4_N_2_)(H_2_O)]
*M* *_r_* = 757.13
Triclinic, 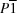

*a* = 8.9080 (3) Å
*b* = 10.7550 (4) Å
*c* = 11.9530 (6) Å
α = 63.446 (1)°
β = 89.364 (2)°
γ = 73.408 (1)°
*V* = 972.60 (7) Å^3^
*Z* = 1
Mo *K*α radiation
μ = 0.09 mm^−1^
*T* = 296 K
0.48 × 0.40 × 0.24 mm

#### Data collection

Bruker APEXII CCD diffractometer
Absorption correction: multi-scan (*SADABS*; Bruker, 2007 ▶) *T*_min_ = 0.955, *T*_max_ = 0.978
11765 measured reflections
3792 independent reflections
3307 reflections with *I* > 2σ(*I*)
*R*_int_ = 0.023

#### Refinement

*R*[*F* ^2^ > 2σ(*F* ^2^)] = 0.039
*wR*(*F* ^2^) = 0.103
*S* = 1.07
3792 reflections
268 parameters
H-atom parameters constrained
Δρ_max_ = 0.22 e Å^−3^
Δρ_min_ = −0.54 e Å^−3^

### 

Data collection: *APEX2* (Bruker, 2007 ▶); cell refinement: *SAINT* (Bruker, 2007 ▶); data reduction: *SAINT*; program(s) used to solve structure: *SIR2004* (Burla *et al.*, 2005 ▶); program(s) used to refine structure: *SHELXL97* (Sheldrick, 2008 ▶); molecular graphics: *ORTEPIII* (Burnett & Johnson, 1996 ▶) and *ORTEP-3 for Windows* (Farrugia, 2012 ▶); software used to prepare material for publication: *SHELXL97*.

## Supplementary Material

Crystal structure: contains datablock(s) I, global. DOI: 10.1107/S1600536812049434/zs2246sup1.cif

Structure factors: contains datablock(s) I. DOI: 10.1107/S1600536812049434/zs2246Isup2.hkl

Additional supplementary materials:  crystallographic information; 3D view; checkCIF report

## Figures and Tables

**Table 1:** Hydrogen-bond geometry (Å, °) *Cg*12 and *Cg*14 are the centroids of the N2/C6–C9 C17–C22 rings, respectively.

*D*—H⋯*A*	*D*—H	H⋯*A*	*D*⋯*A*	*D*—H⋯*A*
C12—H12⋯*Cg*12^i^	0.93	2.97	3.8860 (19)	168
C14—H14⋯*Cg*14^ii^	0.93	2.70	3.584 (2)	159
C21—H21⋯*Cg*12^iii^	0.93	2.85	3.6240 (18)	141

Symmetry codes: (i) 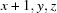; (ii) 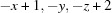; (iii) 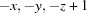.

**Fig. 1.**
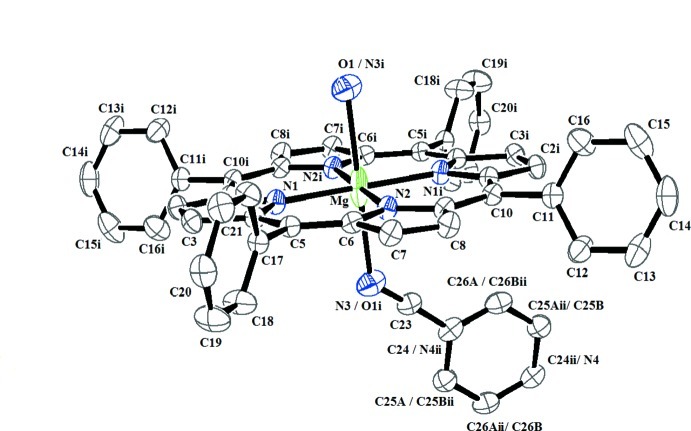
A view of the structure of the complex [Mg(C44H28N4)(C6H4N2)(H2O)] showing the atom numbering scheme. Displacement ellipsoids are drawn at the 40% level. The H atoms have been omitted for clarity. For symmetry codes: (i) -x, -y + 1, -z + 1; (ii) (i) -x + 1, -y, -z + 1.

**Fig. 2.**
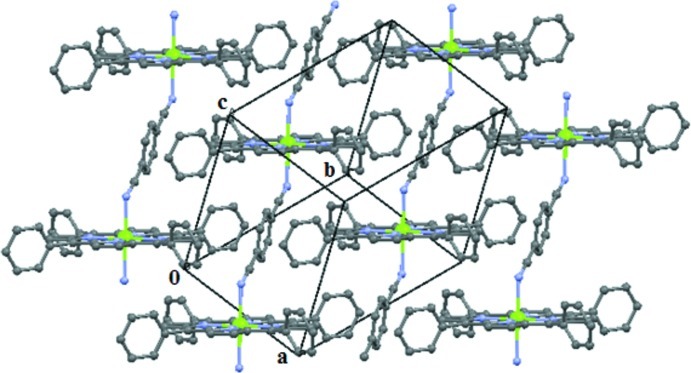
Part of the crystal structure showing weak C—H······π intermolecular interactions.

## supplementary crystallographic information

### Comment

The majority of the reported metalloporphyrin structures involve metals from the first-row transition series. However, the structures of magnesium porphyrins are also of interest because of their relationship to chlorophyll. but the Cambridge Structural Database (CSD; Allen, 2002) contains only 24 structures of these. In order to gain more insight into the geometry of the latter species, we report herein the crystal structure of the title complex, [Mg(TPP)(4-CNpy)(H_2_O)] (where TPP is the 5,10,15,20-tetraphenylporphyrin dianion and 4-CNpy is 4-cyanopyridine).

In the title complex the Mg cation is octahedrally coordinated and lies on an inversion center with the axially-located 4-cyanopyridine and aqua ligands having occupancy factors of 0.5. The atoms of these disordered ligands are divided in two parts: in part 1, the fragment containing the atoms N3, C23, C24, C25A and C26A and in part 2, the atoms O1, N4, C25B and C26B, related by the symmetry code (i) -*x*, -*y*+1, -*z*+1. The 4-cyanopyridine ligand also has inversion symmetry with 50% occupancy [symmetry code (ii): -*x*+1, -*y*, -*z*+1] (Fig. 1). The average equatorial Mg—N(pyrrole) bond distance [2.062 (1) Å] is normal for Mg–porphyrin complexes. The Mg—O(H_2_O) bond length [2.3798 (12)Å] is longer than that found in the related complex [Mg(TPP)(H_2_O)] (2.012 (6) Å) (Choon *et al.*, 1986) but is shorter than the one reported for the non-porphyrin complex *catena*-[diaquatetrakis(µ_4_-oxalato)-tris(µ_2_-oxalato)- dimagnesium-dipotassium-diuranium(IV) nonahydrate clathrate] [2.747 (5) Å] (Imaz *et al.*, 2005).

The axial Mg—N(4-CNpy) bond length [2.3798 (12) Å] is longer than that found in the [Mg(TPP)][TCNQF4] complex [2.266 (2) Å] (where TCNQF4 is 2,3,5,6-tetrafluoro-7,7,8,8-tetracyanoquinodimethane) (Hibbs *et al.*, 2003), but is within the range of 2.152 (2)–2.57 (1) Å found for several magnesium-nitrile non-porphyrin complexes in the CSD [refcodes FEGQAJ (Etkin *et al.*, 1998) and SISWUN (Yang *et al.*, 2008) (Allen, 2002).

The crystal structure resembles a one-dimensional coordination polymer where two [Mg(TPP)] moieties are linked by statistically 50% disordered 4-cyanopyridine and H_2_O ligands. The crystal packing of the title compound is stabilized by weak C—H···π intermolecular interactions involving *Cg* pyrrole and phenyl rings (Table 1 and Fig. 2).

### Experimental

To a solution of [Mg(TPP)(H_2_O)] (Timkovich & Tulinsky, 1969) (15 mg, 0.022 mmol) in chlorobenzene (10 ml) was added an excess of 4-cyanopyridine (50 mg, 0.480 mmol). The reaction mixture was stirred at room temperature and at the end of the reaction, the color of the solution gradually changed from purple to blue–purple. Crystals of the title complex were obtained by diffusion of n-hexane through the chlorobenzene solution.

### Refinement

The H atoms of the statistically disordered aqua ligand were not consideredted. and all H atoms attached to C atoms were fixed geometrically and treated as riding with C—H = 0.93 Å and with *U*_iso_(H) = 1.2*U*_eq_(C). The H atoms of the water molecule were not located

### Crystal data

**Table:**

[Mg(C_44_H_28_N_4_)(C_6_H_4_N_2_)(H_2_O)]	*Z* = 1
*M**_r_* = 757.13	*F*(000) = 394.0
Triclinic, *P*1	*D*_x_ = 1.293 Mg m^−^^3^
Hall symbol: -P 1	Mo *K*α radiation, λ = 0.71073 Å
*a* = 8.9080 (3) Å	Cell parameters from 6453 reflections
*b* = 10.7550 (4) Å	θ = 2.7–27.9°
*c* = 11.9530 (6) Å	µ = 0.09 mm^−^^1^
α = 63.446 (1)°	*T* = 296 K
β = 89.364 (2)°	Block, purple
γ = 73.408 (1)°	0.48 × 0.40 × 0.24 mm
*V* = 972.60 (7) Å^3^	

### Data collection

**Table:**

Bruker APEXII CCD diffractometer	3792 independent reflections
Radiation source: fine-focus sealed tube	3307 reflections with *I* > 2σ(*I*)
Graphite monochromator	*R*_int_ = 0.023
φ and ω scans	θ_max_ = 26.0°, θ_min_ = 1.9°
Absorption correction: multi-scan (*SADABS*; Bruker, 2007)	*h* = −8→10
*T*_min_ = 0.955, *T*_max_ = 0.978	*k* = −13→13
11765 measured reflections	*l* = −14→14

### Refinement

**Table:**

Refinement on *F*^2^	Primary atom site location: structure-invariant direct methods
Least-squares matrix: full	Secondary atom site location: difference Fourier map
*R*[*F*^2^ > 2σ(*F*^2^)] = 0.039	Hydrogen site location: inferred from neighbouring sites
*wR*(*F*^2^) = 0.103	H-atom parameters constrained
*S* = 1.07	*w* = 1/[σ^2^(*F*_o_^2^) + (0.0446*P*)^2^ + 0.2998*P*] where *P* = (*F*_o_^2^ + 2*F*_c_^2^)/3
3792 reflections	(Δ/σ)_max_ = 0.001
268 parameters	Δρ_max_ = 0.22 e Å^−^^3^
0 restraints	Δρ_min_ = −0.54 e Å^−^^3^

### Special details

**Table:**

Geometry. All s.u.'s (except the s.u. in the dihedral angle between two l.s. planes) are estimated using the full covariance matrix. The cell s.u.'s are taken into account individually in the estimation of s.u.'s in distances, angles and torsion angles; correlations between s.u.'s in cell parameters are only used when they are defined by crystal symmetry. An approximate (isotropic) treatment of cell s.u.'s is used for estimating s.u.'s involving l.s. planes.
Refinement. Refinement of *F*^2^ against ALL reflections. The weighted *R*-factor *wR* and goodness of fit *S* are based on *F*^2^, conventional *R*-factors *R* are based on *F*, with *F* set to zero for negative *F*^2^. The threshold expression of *F*^2^ > σ(*F*^2^) is used only for calculating *R*-factors(gt) *etc*. and is not relevant to the choice of reflections for refinement. *R*-factors based on *F*^2^ are statistically about twice as large as those based on *F*, and *R*- factors based on ALL data will be even larger.

### Fractional atomic coordinates and isotropic or equivalent isotropic displacement parameters (Å^2^)

**Table:**

	*x*	*y*	*z*	*U*_iso_*/*U*_eq_	Occ. (<1)
Mg	0.0000	0.5000	0.5000	0.0558 (3)	
N1	0.12116 (13)	0.31528 (11)	0.66326 (11)	0.0307 (3)	
N2	−0.15077 (13)	0.39306 (11)	0.48540 (10)	0.0289 (2)	
C1	0.25095 (15)	0.29941 (14)	0.73443 (12)	0.0291 (3)	
C2	0.30581 (17)	0.15246 (14)	0.83701 (13)	0.0327 (3)	
H2	0.3920	0.1139	0.8989	0.039*	
C3	0.20800 (17)	0.08127 (14)	0.82614 (13)	0.0320 (3)	
H3	0.2143	−0.0154	0.8793	0.038*	
C4	0.09273 (15)	0.18314 (13)	0.71703 (12)	0.0286 (3)	
C5	−0.02834 (15)	0.15157 (13)	0.66983 (12)	0.0279 (3)	
C6	−0.14087 (15)	0.25020 (13)	0.56257 (12)	0.0284 (3)	
C7	−0.26537 (16)	0.21639 (15)	0.51694 (14)	0.0336 (3)	
H7	−0.2849	0.1269	0.5526	0.040*	
C8	−0.34809 (16)	0.33799 (15)	0.41330 (14)	0.0342 (3)	
H8	−0.4355	0.3485	0.3642	0.041*	
C9	−0.27529 (15)	0.44944 (14)	0.39274 (13)	0.0292 (3)	
C10	0.32299 (15)	0.40857 (14)	0.70979 (13)	0.0294 (3)	
C11	0.45965 (16)	0.37258 (14)	0.80258 (13)	0.0312 (3)	
C12	0.61223 (18)	0.34910 (18)	0.77378 (16)	0.0457 (4)	
H12	0.6319	0.3527	0.6960	0.055*	
C13	0.7370 (2)	0.32017 (19)	0.85992 (19)	0.0557 (5)	
H13	0.8394	0.3047	0.8393	0.067*	
C14	0.7102 (2)	0.31430 (17)	0.97470 (17)	0.0544 (5)	
H14	0.7938	0.2953	1.0321	0.065*	
C15	0.5591 (3)	0.3367 (2)	1.00457 (16)	0.0604 (5)	
H15	0.5402	0.3322	1.0828	0.073*	
C16	0.4342 (2)	0.36610 (19)	0.91893 (15)	0.0484 (4)	
H16	0.3321	0.3816	0.9401	0.058*	
C17	−0.03842 (15)	−0.00131 (14)	0.73710 (12)	0.0286 (3)	
C18	−0.1134 (2)	−0.04637 (17)	0.84312 (16)	0.0474 (4)	
H18	−0.1529	0.0167	0.8777	0.057*	
C19	−0.1303 (2)	−0.18509 (18)	0.89874 (17)	0.0546 (5)	
H19	−0.1808	−0.2143	0.9703	0.065*	
C20	−0.07258 (19)	−0.27960 (16)	0.84860 (15)	0.0423 (4)	
H20	−0.0866	−0.3714	0.8846	0.051*	
C21	0.00540 (19)	−0.23746 (15)	0.74544 (14)	0.0402 (3)	
H21	0.0466	−0.3015	0.7122	0.048*	
C22	0.02310 (18)	−0.09931 (15)	0.69032 (13)	0.0355 (3)	
H22	0.0773	−0.0721	0.6207	0.043*	
N3	0.17377 (15)	0.40333 (15)	0.38534 (13)	0.0541 (3)	0.50
C23	0.2697 (3)	0.2742 (3)	0.4230 (3)	0.0370 (6)	0.50
C24	0.38443 (16)	0.13429 (15)	0.45960 (14)	0.0406 (3)	0.50
C25A	0.43163 (19)	0.04190 (18)	0.58443 (17)	0.0465 (4)	0.50
H25A	0.3859	0.0692	0.6437	0.056*	0.50
C26A	0.45348 (19)	0.09187 (17)	0.37545 (16)	0.0436 (4)	0.50
H26A	0.4230	0.1538	0.2894	0.052*	0.50
O1	0.17377 (15)	0.40333 (15)	0.38534 (13)	0.0541 (3)	0.50
C25B	0.43163 (19)	0.04190 (18)	0.58443 (17)	0.0465 (4)	0.50
H25B	0.3859	0.0692	0.6437	0.056*	0.50
C26B	0.45348 (19)	0.09187 (17)	0.37545 (16)	0.0436 (4)	0.50
H26B	0.4230	0.1538	0.2894	0.052*	0.50
N4	0.38443 (16)	0.13429 (15)	0.45960 (14)	0.0406 (3)	0.50

### Atomic displacement parameters (Å^2^)

**Table:**

	*U*^11^	*U*^22^	*U*^33^	*U*^12^	*U*^13^	*U*^23^
Mg	0.0624 (5)	0.0257 (4)	0.0585 (5)	−0.0250 (3)	−0.0308 (4)	0.0055 (3)
N1	0.0329 (6)	0.0214 (5)	0.0355 (6)	−0.0107 (5)	−0.0004 (5)	−0.0098 (5)
N2	0.0306 (6)	0.0205 (5)	0.0339 (6)	−0.0090 (4)	0.0014 (5)	−0.0103 (5)
C1	0.0312 (7)	0.0237 (6)	0.0315 (7)	−0.0080 (5)	0.0023 (5)	−0.0125 (5)
C2	0.0379 (7)	0.0262 (7)	0.0306 (7)	−0.0092 (6)	−0.0013 (6)	−0.0106 (6)
C3	0.0408 (8)	0.0219 (6)	0.0301 (7)	−0.0105 (6)	0.0026 (6)	−0.0090 (5)
C4	0.0326 (7)	0.0216 (6)	0.0318 (7)	−0.0093 (5)	0.0063 (5)	−0.0121 (5)
C5	0.0312 (7)	0.0224 (6)	0.0317 (7)	−0.0103 (5)	0.0074 (5)	−0.0127 (5)
C6	0.0299 (7)	0.0228 (6)	0.0352 (7)	−0.0112 (5)	0.0076 (5)	−0.0141 (6)
C7	0.0333 (7)	0.0256 (7)	0.0430 (8)	−0.0143 (6)	0.0042 (6)	−0.0137 (6)
C8	0.0314 (7)	0.0289 (7)	0.0430 (8)	−0.0134 (6)	0.0013 (6)	−0.0147 (6)
C9	0.0285 (7)	0.0250 (6)	0.0357 (7)	−0.0094 (5)	0.0038 (5)	−0.0149 (6)
C10	0.0294 (7)	0.0251 (6)	0.0336 (7)	−0.0084 (5)	0.0027 (5)	−0.0137 (6)
C11	0.0364 (7)	0.0207 (6)	0.0353 (7)	−0.0104 (5)	−0.0002 (6)	−0.0111 (6)
C12	0.0351 (8)	0.0512 (9)	0.0553 (10)	−0.0066 (7)	0.0015 (7)	−0.0325 (8)
C13	0.0335 (8)	0.0517 (10)	0.0795 (13)	−0.0031 (7)	−0.0095 (8)	−0.0341 (10)
C14	0.0607 (11)	0.0328 (8)	0.0578 (11)	−0.0146 (8)	−0.0252 (9)	−0.0103 (8)
C15	0.0873 (15)	0.0642 (12)	0.0346 (9)	−0.0411 (11)	−0.0020 (9)	−0.0162 (8)
C16	0.0549 (10)	0.0589 (10)	0.0407 (9)	−0.0323 (8)	0.0104 (7)	−0.0224 (8)
C17	0.0307 (7)	0.0229 (6)	0.0311 (7)	−0.0108 (5)	0.0024 (5)	−0.0101 (5)
C18	0.0646 (11)	0.0353 (8)	0.0525 (10)	−0.0237 (8)	0.0282 (8)	−0.0246 (8)
C19	0.0731 (12)	0.0412 (9)	0.0552 (10)	−0.0326 (9)	0.0328 (9)	−0.0190 (8)
C20	0.0516 (9)	0.0259 (7)	0.0459 (9)	−0.0199 (7)	0.0004 (7)	−0.0090 (6)
C21	0.0512 (9)	0.0269 (7)	0.0447 (8)	−0.0114 (6)	0.0011 (7)	−0.0188 (7)
C22	0.0448 (8)	0.0293 (7)	0.0337 (7)	−0.0133 (6)	0.0081 (6)	−0.0148 (6)
N3	0.0445 (7)	0.0621 (9)	0.0702 (9)	−0.0178 (6)	0.0123 (6)	−0.0423 (8)
C23	0.0330 (14)	0.0408 (16)	0.0484 (17)	−0.0179 (13)	0.0103 (12)	−0.0266 (14)
C24	0.0332 (7)	0.0348 (7)	0.0597 (9)	−0.0124 (6)	0.0090 (6)	−0.0258 (7)
C25A	0.0426 (9)	0.0494 (9)	0.0586 (10)	−0.0149 (7)	0.0168 (8)	−0.0342 (9)
C26A	0.0428 (9)	0.0408 (8)	0.0469 (9)	−0.0141 (7)	0.0085 (7)	−0.0196 (7)
O1	0.0445 (7)	0.0621 (9)	0.0702 (9)	−0.0178 (6)	0.0123 (6)	−0.0423 (8)
C25B	0.0426 (9)	0.0494 (9)	0.0586 (10)	−0.0149 (7)	0.0168 (8)	−0.0342 (9)
C26B	0.0428 (9)	0.0408 (8)	0.0469 (9)	−0.0141 (7)	0.0085 (7)	−0.0196 (7)
N4	0.0332 (7)	0.0348 (7)	0.0597 (9)	−0.0124 (6)	0.0090 (6)	−0.0258 (7)

### Geometric parameters (Å, º)

**Table:**

Mg—N2^i^	2.0552 (10)	C12—C13	1.391 (2)
Mg—N2	2.0552 (10)	C12—H12	0.9300
Mg—N1^i^	2.0678 (11)	C13—C14	1.366 (3)
Mg—N1	2.0678 (11)	C13—H13	0.9300
Mg—N3	2.3798 (12)	C14—C15	1.371 (3)
Mg—O1^i^	2.3798 (12)	C14—H14	0.9300
Mg—N3^i^	2.3798 (12)	C15—C16	1.386 (2)
N1—C1	1.3670 (17)	C15—H15	0.9300
N1—C4	1.3720 (16)	C16—H16	0.9300
N2—C9	1.3666 (17)	C17—C18	1.380 (2)
N2—C6	1.3700 (16)	C17—C22	1.3835 (18)
C1—C10	1.4121 (18)	C18—C19	1.389 (2)
C1—C2	1.4421 (18)	C18—H18	0.9300
C2—C3	1.3545 (19)	C19—C20	1.377 (2)
C2—H2	0.9300	C19—H19	0.9300
C3—C4	1.4371 (19)	C20—C21	1.367 (2)
C3—H3	0.9300	C20—H20	0.9300
C4—C5	1.4109 (18)	C21—C22	1.3866 (19)
C5—C6	1.4068 (19)	C21—H21	0.9300
C5—C17	1.5029 (17)	C22—H22	0.9300
C6—C7	1.4425 (18)	N3—C23	1.278 (3)
C7—C8	1.345 (2)	C23—C24	1.429 (3)
C7—H7	0.9300	C24—C26A	1.355 (2)
C8—C9	1.4473 (18)	C24—C25A	1.357 (2)
C8—H8	0.9300	C25A—C26A^ii^	1.379 (2)
C9—C10^i^	1.4079 (19)	C25A—H25A	0.9300
C10—C11	1.4962 (18)	C26A—C25A^ii^	1.379 (2)
C11—C12	1.379 (2)	C26A—H26A	0.9300
C11—C16	1.381 (2)		
			
N2^i^—Mg—N2	180.0	N2—C9—C8	109.27 (11)
N2^i^—Mg—N1^i^	89.69 (4)	C10^i^—C9—C8	124.75 (12)
N2—Mg—N1^i^	90.31 (4)	C9^i^—C10—C1	125.94 (12)
N2^i^—Mg—N1	90.31 (4)	C9^i^—C10—C11	116.57 (11)
N2—Mg—N1	89.69 (4)	C1—C10—C11	117.46 (12)
N1^i^—Mg—N1	180.00 (6)	C12—C11—C16	118.37 (14)
N2^i^—Mg—N3	90.58 (4)	C12—C11—C10	121.54 (13)
N2—Mg—N3	89.42 (4)	C16—C11—C10	120.07 (13)
N1^i^—Mg—N3	92.22 (5)	C11—C12—C13	120.64 (16)
N1—Mg—N3	87.78 (5)	C11—C12—H12	119.7
N2^i^—Mg—O1^i^	89.42 (4)	C13—C12—H12	119.7
N2—Mg—O1^i^	90.58 (4)	C14—C13—C12	120.42 (17)
N1^i^—Mg—O1^i^	87.78 (5)	C14—C13—H13	119.8
N1—Mg—O1^i^	92.22 (5)	C12—C13—H13	119.8
N3—Mg—O1^i^	180.0	C13—C14—C15	119.42 (15)
N2^i^—Mg—N3^i^	89.42 (4)	C13—C14—H14	120.3
N2—Mg—N3^i^	90.58 (4)	C15—C14—H14	120.3
N1^i^—Mg—N3^i^	87.78 (5)	C14—C15—C16	120.40 (17)
N1—Mg—N3^i^	92.22 (5)	C14—C15—H15	119.8
N3—Mg—N3^i^	180.0	C16—C15—H15	119.8
O1^i^—Mg—N3^i^	0.00 (5)	C11—C16—C15	120.74 (16)
C1—N1—C4	106.86 (11)	C11—C16—H16	119.6
C1—N1—Mg	126.20 (9)	C15—C16—H16	119.6
C4—N1—Mg	126.85 (9)	C18—C17—C22	118.23 (12)
C9—N2—C6	107.11 (10)	C18—C17—C5	121.67 (12)
C9—N2—Mg	126.19 (9)	C22—C17—C5	120.06 (12)
C6—N2—Mg	126.63 (9)	C17—C18—C19	120.57 (14)
N1—C1—C10	125.41 (12)	C17—C18—H18	119.7
N1—C1—C2	109.50 (11)	C19—C18—H18	119.7
C10—C1—C2	125.07 (12)	C20—C19—C18	120.41 (15)
C3—C2—C1	106.99 (12)	C20—C19—H19	119.8
C3—C2—H2	126.5	C18—C19—H19	119.8
C1—C2—H2	126.5	C21—C20—C19	119.51 (13)
C2—C3—C4	107.15 (12)	C21—C20—H20	120.2
C2—C3—H3	126.4	C19—C20—H20	120.2
C4—C3—H3	126.4	C20—C21—C22	120.09 (13)
N1—C4—C5	125.00 (12)	C20—C21—H21	120.0
N1—C4—C3	109.50 (11)	C22—C21—H21	120.0
C5—C4—C3	125.48 (12)	C17—C22—C21	121.14 (13)
C6—C5—C4	125.88 (12)	C17—C22—H22	119.4
C6—C5—C17	115.96 (11)	C21—C22—H22	119.4
C4—C5—C17	118.15 (11)	C23—N3—Mg	129.10 (16)
N2—C6—C5	125.82 (11)	N3—C23—C24	176.0 (3)
N2—C6—C7	109.15 (11)	C26A—C24—C25A	118.27 (14)
C5—C6—C7	125.04 (12)	C26A—C24—C23	123.09 (18)
C8—C7—C6	107.45 (12)	C25A—C24—C23	118.58 (17)
C8—C7—H7	126.3	C24—C25A—C26A^ii^	120.85 (15)
C6—C7—H7	126.3	C24—C25A—H25A	119.6
C7—C8—C9	107.02 (12)	C26A^ii^—C25A—H25A	119.6
C7—C8—H8	126.5	C24—C26A—C25A^ii^	120.88 (15)
C9—C8—H8	126.5	C24—C26A—H26A	119.6
N2—C9—C10^i^	125.94 (12)	C25A^ii^—C26A—H26A	119.6

Symmetry codes: (i) −*x*, −*y*+1, −*z*+1; (ii) −*x*+1, −*y*, −*z*+1.

### Hydrogen-bond geometry (Å, º)

Cg12 and Cg14 are the centroids of the N2/C6–C9 C17–C22 rings, respectively.

**Table:**

*D*—H···*A*	*D*—H	H···*A*	*D*···*A*	*D*—H···*A*
C12—H12···*Cg*12^iii^	0.93	2.97	3.8860 (19)	168
C14—H14···*Cg*14^iv^	0.93	2.70	3.584 (2)	159
C21—H21···*Cg*12^v^	0.93	2.85	3.6240 (18)	141

Symmetry codes: (iii) *x*+1, *y*, *z*; (iv) −*x*+1, −*y*, −*z*+2; (v) −*x*, −*y*, −*z*+1.
